# Updated CDC Recommendation for Serologic Diagnosis of Lyme Disease

**DOI:** 10.15585/mmwr.mm6832a4

**Published:** 2019-08-16

**Authors:** Paul Mead, Jeannine Petersen, Alison Hinckley

**Affiliations:** 1 Division of Vector-borne Diseases, National Center for Emerging and Zoonotic Infectious Diseases, CDC.

Lyme disease is a tickborne zoonosis for which serologic testing is the principal means of laboratory diagnosis. In 1994, the Association of State and Territorial Public Health Laboratory Directors, CDC, the Food and Drug Administration (FDA), the National Institutes of Health (NIH), the Council of State and Territorial Epidemiologists, and the National Committee for Clinical Laboratory Standards convened the Second National Conference on Serologic Diagnosis of Lyme Disease (*1*).

The conference proceedings recommended a two-test methodology using a sensitive enzyme immunoassay (EIA) or immunofluorescence assay as a first test, followed by a western immunoblot assay for specimens yielding positive or equivocal results (*1*,*2*). Regarding the development of future tests, the report advised that evaluation of new serologic assays include blind testing against a comprehensive challenge panel, and that new assays should only be recommended if their specificity, sensitivity, and precision equaled or surpassed the performance of tests used in the recommended two-test procedure. To assist serologic test developers, CDC has made available, with support from NIH, a comprehensive panel of sera from patients with various stages of Lyme disease and other conditions, as well as healthy persons (*3*). 

On July 29, 2019, FDA cleared several Lyme disease serologic assays with new indications for use based on a modified two-test methodology (*4*). The modified methodology uses a second EIA in place of a western immunoblot assay. Clearance by FDA of the new Lyme disease assays indicates that test performance has been evaluated and is “substantially equivalent to or better than” a legally marketed predicate test.

## Recommendation

When cleared by FDA for this purpose, serologic assays that utilize EIA rather than western immunoblot assay in a two-test format are acceptable alternatives for the laboratory diagnosis of Lyme disease. Based on the criteria established at the 1994 Second National Conference on Serologic Diagnosis of Lyme Disease, clinicians and laboratories should consider serologic tests cleared by FDA as CDC-recommended procedures for Lyme disease serodiagnosis.

**Summary**

**What is already known about this topic?**

Serologic testing is the principal means of laboratory diagnosis of Lyme disease. Current recommendations include using a sensitive enzyme immunoassay (EIA) or immunofluorescence assay, followed by a western immunoblot assay for specimens yielding positive or equivocal results.

**What is added by this report?**

On July 29, 2019, the Food and Drug Administration (FDA) cleared several Lyme disease serologic assays with new indications for use, allowing for an EIA rather than western immunoblot assay as the second test in a Lyme disease testing algorithm.

**What are the indications for public health practice?**

When cleared by FDA for this purpose, serologic assays that utilize a second EIA in place of western immunoblot assay are acceptable alternatives for the serologic diagnosis of Lyme disease.
